# The Effect of Commercially Available Sugar Alternatives on *Bacillus* Probiotic Viability During Baking

**DOI:** 10.1155/ijfo/9961176

**Published:** 2025-07-01

**Authors:** Jessie Payne, Brooke Holt, Danielle Bellmer, Destiny Wahweah

**Affiliations:** ^1^Department of Animal and Food Science, Oklahoma State University, Stillwater, Oklahoma, USA; ^2^Robert M. Kerr Food and Agricultural Products Center, Oklahoma State University, Stillwater, Oklahoma, USA; ^3^Department of Horticulture Landscape Architecture, Oklahoma State University, Stillwater, Oklahoma, USA; ^4^Department of Biosystems and Agricultural Engineering, Oklahoma State University, Stillwater, Oklahoma, USA

**Keywords:** artificial sweeteners, *Bacillus*, *Bacillus subtilis*, *Lactobacillus*, probiotics, sugar alternatives

## Abstract

Growing health concerns regarding sucrose consumption have led to an increased use of alternative sugars, including sugar alcohols, artificial sweeteners, and natural sweeteners. This study investigated the impact of commercially available sugar substitutes on the viability of *Bacillus* probiotics and *Lactobacillus* strains during baking. Our findings revealed that *Bacillus subtilis* strains exhibited the highest log reduction with sucralose (average 0.99 log CFU/g) and the lowest with monk fruit (average 0.60 log CFU/g). In contrast, *Lactobacillus acidophilus* demonstrated a higher log reduction, with the highest reduction observed with monk fruit (4.18 log CFU/g) and the lowest with sucralose (3.47 log CFU/g). Notably, *B. subtilis* strains exhibited significantly greater viability during baking compared to *L. acidophilus* (*p* < 0.05). Furthermore, *Bacillus* probiotics maintained their viability even under high-temperature baking conditions, suggesting their potential for use in baked goods as a viable probiotic ingredient. Additionally, the use of sugar alternatives, such as monk fruit, sucralose, and stevia, was found to significantly increase the water activity in baked cookies, which may contribute to decreased stability and preservation of probiotic efficacy. This study underscores the superior stability of *Bacillus* probiotics in baked products and highlights the potential benefits of using sugar substitutes to enhance both product shelf life and health benefits.

## 1. Introduction

Table sugar, commonly referred to as sucrose, is a simple carbohydrate widely used as a sweetener in food and beverage products. Sugar replacements such as artificial and natural sweeteners have been developed as many consumers are looking for lower calorie, zero sugar, and “healthier” replacements for table sugar. Not only this, but those with medical conditions such as diabetes mellitus that cannot regulate glucose need to reduce their carbohydrate and sugar intake, but these individuals still want to eat the foods they love. Additionally, health concerns regarding the impact of sucrose consumption within the diet have increased. In response, the American Diabetes Association (ADA) advises reducing daily calorie intake, limiting carbohydrate consumption, and incorporating nonnutritive sweeteners to support a healthy eating pattern and promote weight loss [1]. In recent years, consumer demand for sugar replacements has greatly increased. The profit margin for the food industry is growing, including the opportunity to use new and “improved” sugar substitutions. Consequently, the alternative sugar sector is projected to grow by 7.2% between 2022 and 2029 [2].

Sugar substitutes are now prevalent in diet sodas, chewing gum, low-sugar ice creams, and puddings. Sweeteners are characterized based on composition. The main characterizations include artificial sweeteners, sugar alcohols, and novel sweeteners. There are six artificial sweeteners approved by both the FDA (Food and Drug Administration) and the EFSA (European Food Safety Authority): saccharin, acesulfame K, aspartame, neotame, advantame, and sucralose [3]. Additionally, there are six sugar alcohols used as sugar substitutes: erythritol, isomalt, lactitol, maltitol, sorbitol, and xylitol. Lastly, four main novel sweeteners are derived from natural sources and are “generally regarded as safe” by the FDA: allulose, monk fruit, stevia, and tagatose [3].

Most recently, a statement was put out by the World Health Organization (WHO) warning against using artificial sweeteners as they could pose undesirable effects from long-term consumption, such as increased risk of contracting Type 2 diabetes, cardiovascular diseases, and even mortality in adults [4]. Due to this, novel sweeteners such as monk fruit and stevia have become a much better sugar substitute option. Monk fruit and stevia are two of the four FDA-approved novel sweeteners [5, 6].

It is believed that certain sugars and artificial sweeteners have varying effects on probiotic viability. While some may negatively impact probiotics, others exhibit prebiotic properties—nondigestible ingredients that support the growth of beneficial gut microbes and improve the health of the host [7]. For instance, stevia has been reported to possess prebiotic characteristics [8], and other sugar substitutes and novel sweeteners, such as monk fruit and sugar alcohols, have also shown prebiotic potential.

Recently, there has been a surge in consumer demand for reduced sugar and sugar-free products, as well as probiotic-containing foods due to their digestive health benefits [9]. Despite this growing interest, the effects of sugar alternatives on probiotics remain underexplored. *Bacillus* is a newly recognized and generally recognized as safe (GRAS) probiotic with no existing literature regarding its viability with alternative sweeteners. Additionally, only a limited number of studies have investigated this issue with well-established probiotics like *Lactobacillus* and *Bifidobacterium* [10–13].

Research indicates that replacing sucrose with stevia does not adversely affect probiotic viability in food products [10–13]. Similarly, neotame, sucralose, and aspartame have been found not to impair the viability of *Lactobacillus* and *Bifidobacterium* probiotic strains [11]. When evaluated, sugar alcohols such as xylitol, maltitol, and isomalt also demonstrated comparable results, indicating no negative impact on probiotics [10, 12, 14]. However, when tested in yogurt, erythritol was found to reduce probiotic survival significantly [10].

Probiotic growth can not only be influenced by carbohydrates but can also be affected by water activity (*a*_w_) that occurs when substituting sugar with alternative sweeteners. *a*_w_ measures the amount of free water available for microbial growth, and research has shown that it is a critical factor affecting probiotic stability in food products [15–17]. Due to their *a*_w_ levels, many baked goods are prone to yeast and mold contamination, resulting in a shortened shelf life. Thus, controlling *a*_w_ is essential for maintaining the texture, structure, and stability of various food products, particularly those that are crunchy, powdered, or dehydrated. Sucrose, with its small molecular size, effectively reduces *a*_w_ by binding with water molecules. The extent of the binding ability depends on the type of sweetener and its molecular form. However, many current sugar substitutes do not reduce *a*_w_ in the same way. Therefore, it is crucial to thoroughly investigate the impact of sugar alternatives on *a*_w_ before replacing sucrose in food products. Despite its importance, there is limited knowledge regarding the stability and changes in the *a*_w_ of food containing different sugar substitutes.

This study is aimed at evaluating the viability of *Bacillus* probiotic strains in cookies made with various commercially available sugar alternatives. Specifically, the primary objective is to compare the effects of sucralose, stevia, and monk fruit with sucrose on the viability of *Bacillus* spores in cookies.

## 2. Materials and Methods

**Figure 1:**
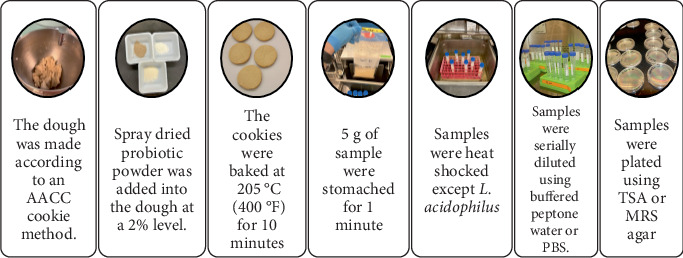
Baking and enumeration process of probiotic cookies.

Cookies were used as the test matrix to investigate the effects of sweeteners on *a*_w_ and probiotic viability.  Figure 1 illustrates the methodology used for the baking and enumeration processes.

### 2.1. Baking Process

Baking was performed using a Frigidaire 30-inch, 4-element, 5.3-cu ft freestanding electric range (Frigidaire, Charlotte, North Carolina, United States). The baking process followed a modified version of the AACC method 10-54.01 for cookie flour baking quality using the microwire-cut formulation [18].

Control cookies were prepared with a standard recipe that included all-purpose flour, granulated sugar, shortening, deionized water, corn syrup, sodium chloride, sodium bicarbonate (baking soda), nonfat dry milk, ammonium bicarbonate (baking powder), and spray-dried probiotic powder ([18]; see Table 1). The preparation involved creaming together the shortening, sugar, nonfat dry milk, sodium chloride, ammonium bicarbonate, and sodium bicarbonate for 3 min. Corn syrup and water were then added and mixed for an additional 2 min. 1.6 g of probiotic powder (11 log CFU/g of *Bacillus subtilis 1* and 10 log CFU/g of *Lactobacillus acidophilus* and *B. subtilis ProSilience HU58*) was incorporated into the flour, which was then mixed into the batter for 2 min. The dough was rolled out and cut into circular shapes with a 60-mm-diameter cookie cutter. Then, the cookies were baked for 10 min at 205°C (400°F). After baking, the cookies were cooled for 10 min before probiotic enumeration.

For cookies with sugar replacements, the same ingredients as the control were used, except that granulated sugar was substituted with one of the following sugar alternatives: stevia, sucralose, or monk fruit (see Table 1). The mixing and baking procedures were identical to those used for the control cookies.

#### 2.1.1. Cookie Samples

In this study, three sugar alternatives were evaluated in cookie formulations alongside a control made with sucrose (see Table 2). The selected sugar substitutes—Lakanto Classic Monk Fruit Sweetener (Lakanto, Orem, Utah, United States), Great Value Sweetener Made with Sucralose (Walmart, Bentonville, Arkansas, United States), and Stevia in the Raw (In the Raw, Cumberland Packing Corp., Brooklyn, New York, United States)—were chosen based on their type and commercial availability in common grocery stores. These alternatives were selected for their 1:1 cup-for-cup volume replacement ratio with granulated sugar.

To ensure accurate substitution, since all alternative sweeteners claimed a 1:1 cup volume ratio, a cup of sucrose was weighed, and the equivalent weight of each sweetener was calculated to replace the sucrose. Table 1 details the ingredients used for each cookie formulation on a weight basis. The sweetening power of these sugar replacements has been noted to not be equal [19], but all commercially available sugar alternatives were claimed by their manufacturers to provide sweetness equivalent to that of sucrose.

### 2.2. Probiotic Strains

Table 3 provides a list of the spray-dried probiotic strains evaluated in this study. All strains were sourced from their respective manufacturing companies. Each probiotic strain used in this study is classified as GRAS. Specifically, *B. subtilis 1* is GRAS-certified by the FDA [20], *L. acidophilus* is GRAS-certified by HoneyCombs Industries, and *B. subtilis ProSilience HU58* is GRAS-certified by Soni & Associates Inc. (GRAS 2012). The growth and spray-drying processes for these probiotic strains were conducted at the respective manufacturing facilities. Due to proprietary considerations, additional methodological details are available upon direct inquiry with the respective companies.

#### 2.2.1. Enumeration of Probiotic Strains

Enumeration protocols were based on the methodology described by Payne et al. [21]. Subsequently, 5.0 g of cookie dough or baked cookies was placed in stomacher bags with 95 mL of either 0.1% sterile-buffered peptone water (for *B. subtilis 1* and *L. acidophilus*) or 0.1 M phosphate-buffered saline (for *HU58*). The samples were then homogenized in a stomacher for 60 s using an EasyMIX Blender (bioMerieux).

For the enumeration process, the *B. subtilis 1* sample was subjected to heat shock at 68°C ± 2°C for 20 min, following the protocol outlined by Jafari et al. [22]. *HU58* samples were heat-shocked at 80°C ± 2°C for 10 min, following the Novonesis enumeration guidelines. *L. acidophilus* was not heat-shocked to avoid significant reductions in cell counts, as noted by De Angelis et al. [23].

Following heat shock, samples containing *B. subtilis 1* and *L. acidophilus* were further diluted in sterile-buffered peptone water and plated using the pour plate method. Samples containing HU58 were diluted in 0.1 M phosphate-buffered saline and plated using the spread plate method. Incubation conditions for each probiotic are detailed in Table 3.

### 2.3. *a*_w_

*a*_w_ was measured using a Neutec Group Inc. LabSwift-aw *a*_w_ meter (Novasina AG, Lachen, Switzerland). Two independent samples were assessed for each treatment group: control, stevia, sucralose, and monk fruit. This procedure was conducted across three independent experiments, resulting in six measurements per treatment. The average *a*_w_ and standard deviation for each product were calculated based on these measurements.

### 2.4. Data Analysis

Data are presented as the average of three independent experiments. The number of log reductions (log(*N*_0_/*N*)) was calculated using the logarithmic difference between the initial dough count before baking (*N*_0_) and the final count in the cookie after baking (*N*). Statistical analysis was performed using SAS 3.81 Enterprise Edition (SAS Institute Inc., Cary, North Carolina, United States). The PROC GLM procedure was used for the analysis of variance, and Tukey's HSD test was applied for mean comparisons to identify significant differences between treatments at a confidence level of *p* < 0.05.

## 3. Results

### 3.1. Impact of Baking Time and Temperature on Probiotic Viability

To evaluate the impact of baking on the viability of probiotics, this study investigated the survival of various probiotic microorganisms under high-temperature baking conditions [21]. Probiotics were exposed to a baking temperature of 205°C for durations of 7, 10, or 12 min, with subsequent measurement of viability reductions. The initial probiotic counts varied by strain: *B. subtilis 1* started with 11.58 ± 0.40 log CFU/g, *B. subtilis ProSilience HU58* with 10.03 ± 0.14 log CFU/g, and *L. acidophilus* with 9.72 ± 0.04 log CFU/g.

**Figure 2:**
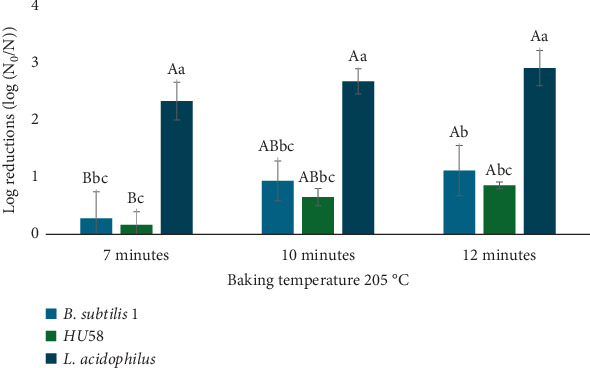
Impact of baking time and temperature on probiotic viability. Note. Capital letters (A, B, C) indicate statistically significant differences (*p* ≤ 0.05) in probiotic viability between baking times. For example, baking times labeled with different capital letters (e.g., A vs. B) are significantly different from one another. Lowercase letters (a, b, c) indicate statistically significant differences (*p* ≤ 0.05) between probiotic strains. That is, strains labeled with different lowercase letters (e.g., a vs. b) differ significantly in their viability.

Figure 2 illustrates the effect of baking at 205°C on probiotic viability, highlighting strain-dependent differences in thermal tolerance. Baking at 205°C resulted in log reductions in viability ranging from 0.17 to 2.92, with statistically significant differences across strains and time points (Figure 2); *B. subtilis* strains showed the lowest losses, likely due to their spore-form state. In contrast, *L. acidophilus* exhibited significantly greater log reductions across all three-time points compared to both *B. subtilis* strains (ANOVA, *p* < 0.05), with the most substantial decline occurring under prolonged baking conditions (see Figure 2 note). While baking time had a significant effect on the viability of *B. subtilis*, no statistically significant time-dependent effect was observed for *L. acidophilus*, indicating distinct thermal response profiles among the strains [21]. These findings are critical for food manufacturers, as maintaining probiotic viability above 6 log CFU is essential for delivering health benefits in baked functional foods.

Although exposed to high temperatures and extended baking durations, surviving populations of *B. subtilis* strains remained at levels likely sufficient to provide health benefits, reflecting their known resilience and potential therapeutic effects. In contrast, the substantial reductions observed in *L. acidophilus* highlight the challenge of maintaining adequate viable probiotic populations in baked goods, especially for less heat-resistant strains. This strain-dependent response is likely due to the spore-forming ability of the *Bacillus* strains, which confers greater thermal resistance. Although *L. acidophilus* viability was significantly reduced (ANOVA, *p* < 0.05), the surviving population remained at approximately 6.1 log CFU/g—just above the commonly cited minimum threshold (6 log CFU/g) necessary to confer health benefits. These findings underscore the importance of selecting probiotic strains with high thermal stability when developing baked probiotic products, as demonstrated in this study (see also [21] for comparable findings).

These findings reinforce the critical importance of maintaining sufficient probiotic counts in baked functional foods and highlight the need to select thermally stable strains or apply protective strategies to preserve probiotic viability postbaking. Such strategies may include formulation optimizations like microencapsulation or matrix buffering, as well as postbaking supplementation to ensure therapeutic benefits. Additional details on the experimental design, including the effects of varying baking temperatures, durations, and other conditions, are available in Payne et al. [21].

### 3.2. Impact of Sugar Replacement on Probiotic Viability

The results provide a comparative analysis of the effects of commercial sugar alternatives versus pure table sugar (sucrose) on probiotic viability (*p* < 0.05). The initial counts ranged from 11.11 to 11.84 ± 0.13 log CFU/g for *B. subtilis 1*, 9.42 to 9.76 ± 0.10 log CFU/g for *L. acidophilus*, and 9.93 to 10.15 ± 0.15 log CFU/g for *HU58*.

**Figure 3:**
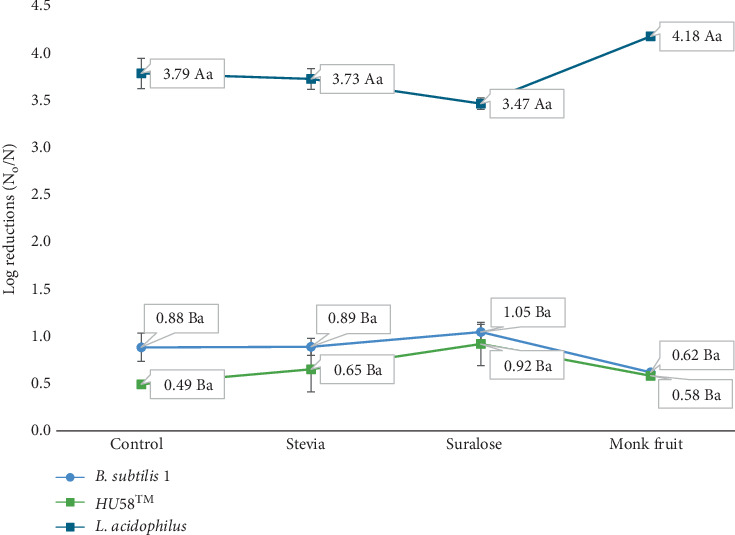
Effect of sugar alternatives on the viability of *Bacillus* probiotics. Note. Different capital letters denote significant differences (*p* ≤ 0.05) between probiotics. Different lowercase letters denote significant differences (*p* ≤ 0.05) among sugars for a single probiotic. Data is presented as the means of individual experiments in triplicate. Initial concentration averages: *B. subtilis 1*: 11.43 log CFU/g, HU58: 10.03 log CFU/g, *L. acidophilus*: 9.62 log CFU/g.

For *B. subtilis* 1, the highest numerical log reductions (1.05 ± 0.08 log CFU/g) were observed when baked with sucralose (Figure 3). Overall, both strains maintained their viability across all tested sugar alternatives, with no statistically significant decline observed (ANOVA, *p* > 0.05). Similarly, for HU58, the greatest log reductions (0.92 ± 0.23 log CFU/g) occurred with sucralose, but again, differences between sugar alternatives were not statistically significant (ANOVA, *p* > 0.05). Overall, both *B. subtilis* strains maintained their viability across all tested sugar alternatives, showing no substantial declines (*p* > 0.05).

In contrast, *L. acidophilus* demonstrated a distinct trend of greater sensitivity to baking conditions and sugar alternatives. Although numerical log reductions were highest when baked with monk fruit (4.18 ± 0.08 log CFU/g) and lowest with sucralose (Figure 3), these differences between sugar alternatives were not statistically significant as shown by shared lowercase letters in Figure 3. Stevia and the control sample were statistically similar to one another. Overall, the sugar alternative did not affect *L. acidophilus*. Nevertheless, *L. acidophilus* experienced a significant decline in viability due to the baking process itself. Notably, even when baked with table sugar (sucrose), viability decreased by over 3 log CFU/g, causing *L. acidophilus* to fall below the required threshold of 6 log CFU/g necessary to confer health benefits (Figure 3) [24].

Figure 3 illustrates the differential impact of various sugar alternatives on the viability of *Bacillus* probiotics compared to *L. acidophilus* (ANOVA, *p* < 0.05). *L. acidophilus* exhibited significantly lower viability than both *B. subtilis* strains across all sugar alternatives, as indicated by different lowercase letters in Figure 3 (*p* < 0.05). In contrast, the two *B. subtilis* strains responded similarly to the sugar alternatives, with no statistically significant differences in viability between them (Tukey's HSD, *p* > 0.05), as shown by shared lowercase letters. This contrast underscores the strain-dependent effects of sugar alternatives on probiotic survival and highlights the need to consider strain-specific probiotic responses when selecting sugar substitutes for baked formulations.

### 3.3. Effect of Sugar Alternatives on *a*_w_

**Figure 4:**
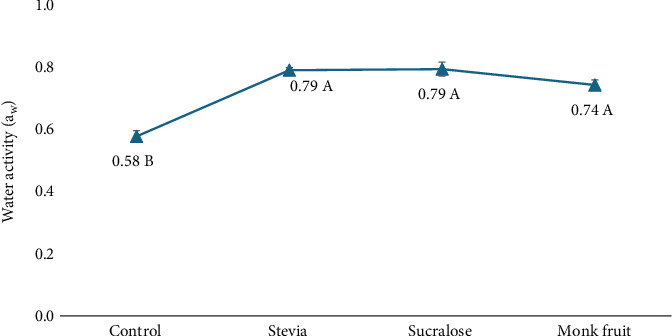
Effect of sugar alternatives on water activity. Note: Different capital letters denote significant differences between treatments (*p* ≤ 0.05) as determined by Tukey HSD. Data are presented as the means of individual experiments in triplicate.

The *a*_w_ of cookies prepared with different sweeteners was measured to assess potential impacts on microbial stability. Typically, cookie *a*_w_ ranges from 0.40 to 0.60 and should remain below 0.70 to effectively limit microbial growth [25, 26]. As shown in Figure 4, the control cookie (made with sucrose) had a *a*_w_ of 0.58, falling within the typical range. In contrast, all cookies made with sugar alternatives exceeded the 0.70 threshold, suggesting a higher potential risk for microbial growth.

The findings reveal that the control cookies (sucrose) had a mean *a*_w_ of 0.58, significantly lower than stevia (0.79), sucralose (0.79), and monk fruit (0.74) (ANOVA, *p* ≤ 0.05). Specifically, cookies made with sugar alternatives exhibited higher *a*_w_ levels (>0.70 *a*_w_), exceeding the threshold for microbial stability and potentially creating a more favorable environment for mold and microbial growth.

Given these results, it is evident that cookies made with alternative sweeteners may require additional measures to enhance their shelf life and prevent microbial contamination. Potential strategies could include incorporating antimicrobial agents (e.g., natural extracts, humectants, and organic acids like acetic, lactic, and propionic acids, as well as essential oils and herbal extracts), modifying formulation ingredients, or adjusting baking conditions to address the increased *a*_w_ associated with sugar replacements. Such adjustments are essential to ensure microbial safety and shelf stability in sugar-reduced cookies.

## 4. Discussion

Probiotic viability is influenced by multiple factors, including *a*_w_, food matrix components (e.g., salt, fat, and carbohydrates), storage conditions, and oxygen exposure [22, 27–30]. The primary objective of this study was to assess the viability of *Bacillus* strains during baking with commercially available alternative sweeteners and to compare their performance to that of *L. acidophilus*, a widely used probiotic in the food industry. *L. acidophilus* served as a benchmark for comparison against the more recently introduced *Bacillus* spores, which are proposed to offer enhanced thermal stability. Initial probiotic counts ranged from 11.11 to 11.84 ± 0.13 log CFU/g for *B. subtilis* 1, 9.42 to 9.76 ± 0.10 log CFU/g for *L. acidophilus*, and 9.93 to 10.15 ± 0.15 log CFU/g for *HU58*. Viability was evaluated based on the ability of each strain to retain at least 6 log CFU/g postbaking, the minimum threshold generally required for health benefits. Cookies were prepared using various sugar alternatives—stevia, sucralose, and monk fruit—substituted at equivalent weights (1:1 cup ratio) to sucrose to ensure consistency in formulation. Sucrose (table sugar) was used as the control.

Research has shown that certain sugars can adversely affect probiotic survival. For example, Shah and Ravula [31] reported that high concentrations of sugar inhibit probiotic growth, while Kimoto-Nira et al. [32] found that fructose, sucrose, and glucose decrease probiotic viability, whereas lactose, xylose, and galactose do not. These effects are strain-dependent, and the impact of sugar alternatives on *Bacillus* probiotics is underexplored. The findings from this study showed that both *Bacillus* probiotics stayed above the required threshold to provide health benefits (average *HU58*, 9.37 log CFU/g, and *B. subtilis 1*, 10.57 log CFU/g) and *L. acidophilus*, on the other hand, was found to fall below the required viability range (5.83 log CFU/g) (Figure 3) [24]. This outcome shows that *L. acidophilus* is not suitable for this application.

The recent GRAS approval of *Bacillus* probiotics, combined with their spore-forming properties, suggests that novel sweeteners with potential prebiotic activity may enhance probiotic viability. Monk fruit, a GRAS-approved, zero-calorie natural sweetener, has gained attention as a functional food ingredient that can replace sugar in reduced-calorie formulations [33]. In addition to its sweetening capability, monk fruit contains bioactive compounds such as mogrosides, which have been associated with antioxidant, anti-inflammatory, and antidiabetic effects—further supporting its classification as a health-promoting natural sweetener. Emerging evidence also suggests that monk fruit may exert prebiotic effects by modulating gut microbiota composition and promoting the growth of beneficial microbes. This raised the hypothesis that monk fruit could support the growth and stability of *Bacillus* strains in baked goods. A prebiotic is defined as a nondigestible fiber that resists digestion in the small intestine and is fermented in the colon, where it is utilized by gut microbiota, thereby conferring health benefits to the host [34, 35].

Current commercial products containing monk fruit, such as Gut Happy, claim to deliver up to 9 log CFU/g of *Bacillus* probiotics. The findings of this study suggest that cookies formulated with monk fruit as a sweetener can support comparable or even higher levels of *Bacillus* probiotic viability than those found in existing products. Among the sweeteners tested, monk fruit supported the highest postbaking viability for the *Bacillus* strains. Notably, monk fruit did not negatively impact *Bacillus* viability overall; however, it was associated with the greatest log reduction in *L. acidophilus*. This reduction may be linked to the presence of erythritol in the monk fruit sweetener blend, as erythritol has previously been shown to impair probiotic survival in yogurt [10]. However, erythritol content was not quantified or controlled in this study.

Stevia has also been claimed to have prebiotic benefits. Previous studies have reported no adverse effects on probiotic viability when stevia replaced sucrose [10–13]. Our study supports these findings, as no significant differences in probiotic viability were observed between stevia and the control for all probiotic strains (Figure 3) (ANOVA, *p* > 0.05).

Research on the effects of nonnutritive sweeteners on probiotics remains limited. Existing studies on *Lactobacillus* and *Bifidobacterium* suggest that sweeteners such as neotame, sucralose, and aspartame do not significantly impair probiotic viability [11]. In our study, no statistically significant differences in *Bacillus* viability were observed between the control (sucrose) and sucralose-treated cookies (ANOVA, *p* > 0.05). However, it is worth noting that cookies containing sucralose showed the highest numerical log reductions for both *Bacillus* strains. While these differences were not statistically significant, they may suggest a trend that warrants further investigation with larger sample sizes or more targeted analysis.

*Bacillus* strains demonstrated greater stability compared to *L. acidophilus*, and the type of sugar substitute did not significantly impact viability. The log reductions among different sweeteners were not statistically significant (ANOVA, *p* > 0.05, e.g., different lowercase letters in Figure 3). Overall, all sugar replacements tested are adequate for use with these strains of *B. subtilis* probiotics.

The second objective of this study was to assess how different sweeteners influence *a*_w_ and their potential impact on microbial stability. Previous research on the effects of alternative sweeteners on *a*_w_ has produced mixed findings. For example, two separate studies by Rubio-Arraez et al. (A and B) have investigated this relationship, highlighting the complexity and variability in outcomes depending on the sweetener type and formulation. In a study by Rubio-Arraez et al. [36], tagatose and isomaltose were used as sucrose substitutes in citrus jelly; *a*_w_ remained stable at 0.98 throughout a 45-day storage period. However, Rubio-Arraez et al. [37] observed a different effect in lemon marmalade, where tagatose was found to lower *a*_w_ similarly to sucrose. Nourmohammadi and Peighambardoust [38] found that replacing sucrose with oligosaccharide, oligofructose, or maltitol led to higher *a*_w_ levels, promoting mold growth within just 3 days. They found that when sucrose was replaced with maltitol or oligofructose, *a*_w_ remained elevated. In another related study, Javanmardi et al. [39] found that substituting sucrose with maltitol significantly increased *a*_w_, while xylitol effectively decreased it. These findings highlight the complex interactions between different alternative sugars and *a*_w_ in baked goods.

The sweetening powers of stevia, monk fruit, and sucralose vary significantly, which can influence both the sweetness profile and the *a*_w_ of baked products (Figure 4, as shown by the different capital letters). These differences in sweetness levels can impact the formulation of baked goods, as the volume of the sweetener used in recipes will be different for stevia, monk fruit, and sucralose compared to sugar. This reduction in bulk may influence the *a*_w_ of the final product. *a*_w_ is crucial for controlling texture, freshness, and microbial stability, and lower levels of bulk sweeteners may result in a product with a higher *a*_w_, potentially affecting its shelf life and moisture retention. Moreover, sugar contributes not only to sweetness but also to the hygroscopic properties of baked goods, aiding in moisture retention and overall texture. Replacing sugar with high-potency sweeteners like stevia, monk fruit, or sucralose often necessitates formulation adjustments to preserve key quality attributes such as texture, moisture balance, and shelf life. These sweeteners are typically used in much smaller quantities due to their intense sweetness and are often blended with bulking agents like maltodextrin to match the volume and functionality of sugar. As a result, adjustments may include modifying the ratio of dry to wet ingredients, incorporating humectants (e.g., glycerin), or adding fiber-based binders to compensate for sugar's structural and moisture-binding roles.

In this study, significant differences in *a*_w_ were observed across the different sweeteners (ANOVA, *p* < 0.05). While previous research has reported that *a*_w_ may not significantly impact the thermal stability of certain probiotic strains during baking (e.g., [21]), our findings similarly indicate that the sugar alternatives tested did not lead to notable changes in probiotic viability. However, cookies formulated with alternative sweeteners exhibited *a*_w_ levels exceeding the recommended threshold of 0.70 *a*_w_ for controlling bacterial growth, with values ranging from 0.74 to 0.79. This elevation in *a*_w_ is a critical consideration, as higher *a*_*w*_ creates a more favorable environment for microbial proliferation, potentially shortening shelf life and raising food safety concerns. These findings highlight the importance of adjusting formulations or incorporating additional preservation strategies when using high-potency sweeteners in baked goods.

Further research stemming from this study has recently been published, specifically examining the sensory acceptability of probiotics in baked goods. Payne et al. [40] conducted consumer sensory evaluations using a 9-point hedonic scale, a 5-point JAR scale, and a ranking scale to assess attributes such as taste, texture, aroma, and overall liking. Results indicated that consumers generally showed a stronger preference for products containing *L. acidophilus* over those with *B. subtilis* strains, particularly in terms of taste and overall acceptance. However, this preference may be influenced by the distinct sensory characteristics of each strain and can potentially be mitigated through the inclusion of flavorings, spices, or other ingredients that mask or complement the flavor profile of the probiotics.

Previous studies have similarly reported that consumer acceptance of *Bacillus* strains can vary significantly depending on the product matrix and formulation. For instance, positive sensory responses have been observed when *Bacillus* strains were incorporated into chocolate, cereal bars, and dairy-based products [41–43]. These findings highlight the importance of the food matrix in shaping consumer perception and underscore the need for future studies to explore matrix–probiotic interactions more thoroughly, particularly in the context of flavor compatibility and textural effects in different food formats.

In future research building on these findings of sugar alternatives, sensory evaluation trials specifically designed for probiotic-enriched cookies should be conducted. These evaluations will assess both the overall consumer acceptability and the sensory attributes (e.g., taste, texture, and aftertaste) of the final product. These studies will inform strategies to incorporate probiotics into sugar-free baked goods while maintaining sensory quality and commercial viability.

In conclusion, this study offers important insights into the viability of *Bacillus* probiotics and *L. acidophilus* in baked products formulated with commercially available sugar alternatives. *Bacillus* strains demonstrated high thermal stability across all sweeteners tested, with no statistically significant differences in viability. In contrast, *L. acidophilus* exhibited reduced viability, particularly in cookies sweetened with monk fruit—a result potentially linked to the presence of erythritol. This finding highlights a need for future research to explore mitigation strategies for erythritol's impact on probiotic survival. Consistent with prior studies, nonnutritive sweeteners such as stevia and sucralose did not significantly impair probiotic viability. However, observed increases in *a*_w_ with alternative sweeteners suggest possible consequences for product shelf life and microbial stability. Further investigations should examine the effects of sugar alcohols like erythritol on probiotic viability and explore consumer acceptance of probiotic-enriched cookies made with reduced sugar formulations.

## 5. Limitations

In the present study, commercial sugar alternatives were evaluated to assess their impact on the stability of *Bacillus* probiotics in cookies. A notable limitation is that the monk fruit sweetener used was not a pure extract but a commercial blend containing erythritol, a sugar alcohol known to influence probiotic viability and sensory attributes. Previous research has shown that erythritol, at certain concentrations, can reduce the survival of probiotics such as *L. acidophilus* in fermented products [10]. Although the exact concentration of erythritol in the monk fruit blend used here was not measured, its presence may have contributed to the observed reductions in probiotic viability, particularly for *L. acidophilus*. To clarify these effects, future studies should use pure monk fruit extract to isolate its specific impact on probiotic stability, thereby eliminating the potential confounding influence of erythritol and other additives.

Additionally, the sensory acceptability of the probiotic-enriched cookies was not assessed in the present study, representing a critical gap in understanding their market viability. Sensory factors—particularly taste, texture, mouthfeel, and aftertaste—can significantly influence consumer perception and acceptance. Prior studies have shown that incorporating probiotics or nonnutritive sweeteners can alter flavor profiles and textural attributes in baked goods, potentially affecting consumer liking and repeat purchase behavior. As discussed earlier, future research should include structured sensory evaluation using consumer panels representative of the target market. Standardized methods such as 9-point hedonic scales for preference testing, combined with purchase intent, would provide actionable insights into consumer acceptance. Evaluating cookies made with different probiotic strains and sugar alternatives in this way will help optimize formulations that balance microbial viability with sensory quality. Ultimately, such studies will be essential for ensuring that functional foods are both beneficial and palatable to a broad audience.

## 6. Conclusion

This study is aimed at evaluating the survival of *Bacillus* probiotic strains in cookies baked with various commercially available sweeteners. The sweeteners tested included Lakanto Classic Monk Fruit Sweetener (monk fruit, primarily mogrosides and erythritol), Great Value Sweetener Made with Sucralose (sucralose), and Stevia in the Raw (stevia, containing steviol glycosides). Results indicated that these alternative sweeteners did not adversely affect probiotic viability in the cookies. Both *B. subtilis* strains demonstrated the highest stability during baking, exhibiting the lowest log reductions in cookies formulated with sweeteners possessing prebiotic properties, such as stevia and monk fruit, as supported by prior studies on their fermentable fiber content (e.g., [33, 34]). In contrast, *L. acidophilus* showed the least stability, particularly when baked with monk fruit, possibly due to the erythritol content, which has been linked to impaired probiotic survival in other matrices [10].

Overall, although some probiotics displayed greater stability, the type of sugar substitute did not significantly impact probiotic viability (ANOVA, *p* > 0.05; see the Results section). A key limitation of this study was the inclusion of erythritol in the monk fruit sweetener, which may have confounded the effects on *L. acidophilus* viability. Additionally, sensory acceptability was not assessed, limiting insights into consumer preference. Future research should explore other sugar alternatives, such as rare sugars tagatose and allulose, which have shown promise due to their prebiotic potential and low glycemic indices, to better understand their influence on probiotic survival and product quality. Integrating sensory evaluations and consumer acceptance studies with probiotic viability assessments will be essential for developing commercially viable functional baked goods that are both health-promoting and well-received by consumers.

## Figures and Tables

**Table 1:** Cookie formulations with alternative sugars.

**Ingredients**	**Control cookie (g)**	**Stevia cookie (g)**	**Sucralose cookie (g)**	**Monk fruit cookie (g)**
Flour	80.0	80.0	80.0	80.0
Sucrose	33.6			
Stevia		5.3		
Sucralose			4.4	
Monk fruit				31.3
Salt	1.0	1.0	1.0	1.0
Sodium bicarbonate	0.4	0.4	0.4	0.4
Ammonium bicarbonate	0.8	0.8	0.8	0.8
Nonfat dry milk	0.8	0.8	0.8	0.8
Shortening	35.0	35.0	35.0	35.0
Corn syrup	1.2	1.2	1.2	1.2
DI water	21.0	21.0	21.0	21.0
Probiotic powder	1.6	1.6	1.6	1.6

**Table 2:** Sweeteners that were used in formulations of cookies.

**Sweetener brand**	**Ingredients**
Great Value Pure Granulated Sugar	Sucrose (table sugar)
Lakanto Classic Monk Fruit Sweetener	Monk fruit extract, erythritol
Great Value Sweetener Made with Sucralose	Sucralose, maltodextrin
Stevia in the Raw	Stevia extract, maltodextrin

**Table 3:** List of strains used as probiotics with their starting concentrations, culture media, and incubation conditions.

**Strain**	**Culture concentration**	**Culture media**	**Incubation conditions**
*Bacillus subtilis* 1	3.0 × 10^11^ CFU g^−1^	Tryptic soy agar (TSA) (g/L: 40 g)	37°C ± 2°C 24 h
*Bacillus subtilis ProSilience HU58*	1.5 × 10^10^ CFU g^−1^	Tryptic soy agar (TSA) (g/L: 40 g)	37°C ± 2°C 24 h
*Lactobacillus acidophilus*	1.2 × 10^10^ CFU g^−1^	De Man, Rogosa, and Sharpe (MRS) agar (g/L: 70 g)	37°C ± 2°C 48 h

## Data Availability

The data supporting this study's findings are available from the corresponding author upon reasonable request.
